# Effects of Low- and High-Frequency Cardiac Rehabilitation on Risk Factors, Physical Fitness and Quality of Life in Middle-Aged Women with Coronary Heart Disease

**DOI:** 10.3390/metabo13040550

**Published:** 2023-04-12

**Authors:** Peng Zhou, Wangyang Zhang, Yonghwan Kim, Huan Meng

**Affiliations:** 1 Department of Physical Education, General Graduate School, Yongin University, Yongin 17092, Republic of Korea; 2 School of Physical Education, Main Campus, Zhengzhou University, Zhengzhou 450001, China; 3 Department of Physical Education, Gangneung-Wonju National University, Gangneung 25457, Republic of Korea

**Keywords:** cardiac rehabilitation, coronary heart disease, low-frequency training, high-frequency training, middle-aged women, fitness, cardiovascular risk factor, quality of life

## Abstract

Cardiac rehabilitation (CR) is a system that comprehensively manages risk factors to reduce the recurrence rate after cardiovascular disease treatment. This study compared the effects of home-based low-frequency CR (1–2 times/week) and center-based high-frequency CR (3–5 times/week) for 12 weeks. This study was conducted as an observational case-control study. Ninety women, ages 45 to 60, who underwent coronary artery stenting were enrolled. Measurement variables were waist circumference, body mass index (BMI), blood pressure (BP), total cholesterol (TC), low-density lipoprotein cholesterol (LDLC), high-density lipoprotein cholesterol (HDLC), triglycerides (TG), glucose, VO_2_ peak, body composition, and quality of life. Significant changes were observed in systolic BP, TC, LDLC, TG, VO_2_ peak, exercise duration, and quality of life in both groups. However, BMI, waist circumference, body fat percentage, HDLC, and blood glucose only exhibited significant changes with HFT. The interaction effects according to time and group were as follows: systolic BP, waist circumference, body fat, BMI, HDLC, and glucose (*p* < 0.05). Therefore, in CR participants, HFT improved more than LFT on obesity factors, HDLC, and glucose change. As well as center-based HFT, home-based LFT also improved risk factors for cardiovascular disease, fitness, and quality of life. For female patients who have difficulty visiting the CR center frequently, home-based LFT may be a CR program that can be presented as an alternative.

## 1. Introduction

Coronary artery disease (CAD) is caused by a narrowing or obstruction of the inner diameter of blood vessels due to plaque buildup in the coronary arteries. Angina pectoris or myocardial infarction are typical manifestations of this condition [1]. CAD has a high incidence and mortality worldwide and is considered an aging-related disease. In recent years, the incidence rate has gradually increased in middle-aged women, and there is the potential for disease recurrence; therefore, continuous management is required after treatment [2,3].

Management methods include monitoring health conditions through regular medical examinations, pharmacotherapy, and participation in structured services provided by the cardiac rehabilitation (CR) system. The CR team is composed of cardiologists, nutritionists, nurses, and clinical exercise specialists, and CR involves exercise, stress management, dietary control, lifestyle modification, smoking cessation, and alcohol restriction [4,5]. In patients with heart disease, CR aims to prevent cardiac events and recurrences, such as acute myocardial infarction, and to aid in the maintenance of physical and mental health by improving obesity, dyslipidemia, hypertension, diabetes, and quality of life [6,7]. Previous studies have revealed that the mortality rate of CR participants can be reduced by up to 53% compared to that of non-participants. Specifically, the cardiac death rate could be lowered by 60% in those participating in continuous CR exercise. Furthermore, major adverse cardiac events can be reduced by 51%, and the recurrence rate of myocardial infarction decreases to 37% [8]. Professional organizations recommend participating in CR exercise 3–5 days a week for cardiovascular disease and recurrence prevention [9,10].

However, despite the positive effects of CR, patients’ participation rate remains low. Reasons for non-participation include patients’ lack of awareness and poor physical health [11,12]. In addition, some factors cannot be improved immediately, such as the time required, cost, distance, and difficult conditions for visiting CR [13,14]. A survey of 297 patients showed that a high participation rate was 1–2 times per week rather than 3–5 times per week [15]. Moreover, women are more likely to be restricted from engaging in CR than men. Experts have reported several cases in which women do not achieve gender equality, such as a lack of social support and awareness of the disease compared to men [16,17].

Research on self-monitoring or CR management using mobile devices has recently been conducted to increase the CR participation rate [18,19,20]. The effectiveness of home-based CR training compared to CR training at specialized centers is equally positive. These CR services provide advantages regarding location movement, cost-effectiveness, and convenience [18,19].

Although there is limited published evidence, researchers agree that low-frequency training positively impacts cardiovascular diseases. In a study involving exercise intervention for 12 weeks, the psychological and mental benefits were greater for participants performing high-frequency CR; however, it was revealed that low-frequency (twice per week) was also beneficial [21]. Other studies reported that quality of life and anaerobic threshold were higher with high-frequency CR, but VO_2_ peak exhibited equivalent improvement with low-frequency CR [22]. Despite such favorable evidence, few studies have been conducted in middle-aged women with cardiovascular disease, and most studies mixed men and women [23,24].

Therefore, this study compared the effects of low-frequency home-based CR and high-frequency center-based CR programs in middle-aged women during early CR following stent implantation. Physical fitness, blood pressure, body composition, blood variables associated with cardiovascular risk factors, and quality of life were measured.

## 2. Materials and Methods

### 2.1. Experimental Procedure and Participants

The sample size calculation, which was performed using the following parameters: effect size dz = 0.5, α err prob = 0.05, power (1-β err prob) = 0.95, revealed that 45 individuals were required for each group. As such, a total of 90 women were enrolled in the two groups. The exercise expert’s time was divided between the group that trained 1–2 days per week (low-frequency training, LFT; *n* = 45) and the group that trained 3–5 days per week (high-frequency training, HFT; *n* = 45) through patient consultation. All participants were middle-aged women (45–60 years) who underwent percutaneous coronary intervention (PCI) with drug-eluting stents at our cardiology department for myocardial infarction, unstable angina, or angina pectoris and were subsequently referred to the CR center. Patients treated with coronary artery bypass grafting or plain angioplasty were excluded from the analysis. After PCI, all patients were treated with lipid regulators, thrombolytic agents, beta-blockers, or calcium channel blockers, and nitroglycerin was prescribed. The first visit for CR was undertaken 4 weeks after discharge. Only patients in the stable phase due to successful cardiac treatment were referred. The pretest was conducted at the first visit, and the posttest was conducted after 12 weeks. During the 12-week exercise intervention period, the patients underwent either home-based training or visited local exercise centers. This study was conducted as an observational case-control study for 12 weeks.

The CR protocol included nursing, nutrition, and exercise management. Participants were provided with a contact number that could be used at any time to access information on the disease, programs, and symptoms during the study period. The researcher carefully explained the study procedure, examination, and publication to all the participants. The study was conducted in accordance with the principles outlined in the Declaration of Helsinki, with the approval of the Institutional Review Board of Asan Medical Center, Seoul (2015-0594). All participants provided written informed consent.

### 2.2. Blood Collection

The factors screened during blood collection were related to cardiovascular disease: total cholesterol (TC), triglycerides (TG), low-density lipoprotein cholesterol (LDLC), high-density lipoprotein cholesterol (HDLC), and glucose. Blood samples were collected after participants fasted for at least 8 h. Blood was drawn primarily from the median cubital vein. Approximately 25–30 mL of blood was collected, placed in an anticoagulant tube, and centrifuged at 3000 rpm for 10 min. The plasma and serum, excluding cell components, were extracted and stored at −70 °C. Lipid and glucose values were obtained using an automatic blood analyzer (Hitachi-747, Tokyo, Japan).

### 2.3. Body Composition and Anthropometry

Bioelectrical impedance analysis (BIA), using Inbody 720 (Inbody Co., Seoul, Republic of Korea) equipment, was used to assess the body composition of participants. The current resistance of the BIA device was measured at 1, 5, 50, 250, 500, and 1000 kHz. The principle underlying BIA is that tissue conducts electricity effectively under conditions of high moisture and low resistance. However, fat increases the resistance to electrical conduction. Therefore, electrical conduction is used to measure the amount of muscle and fat. To minimize water loss before the test, saunas or prolonged bathing were prohibited. The test room temperature was maintained between 20 and 25 °C, and items such as jewelry were removed. Among patients with heart disease, those with a pacemaker were excluded from the examination. In addition, patients who had a cast placed on their hand or leg and were unable to stand owing to inconvenience or discomfort or those with an amputated hand or leg were excluded from the examination. We measured height, weight, body mass index (BMI), body fat percentage, and skeletal muscle percentage.

Waist circumference was measured directly using a measuring tape, horizontally placed 2 cm above the navel. Natural breathing was permitted during the measurement.

### 2.4. Fitness Test

The American College of Sports Medicine (ACSM, Indianapolis, IN, USA) guidelines were followed to assess cardiovascular endurance, and oxygen uptake was evaluated by the graded exercise test (GXT) [25]. The GXT used the Vmax29 exercise stress test equipment (Sensormedics Co., Yorba Linda, CA, USA). An electrocardiogram (ECG) under the supervision of a physician was used to monitor for abnormalities, while oxygen uptake capacity (VO_2_ peak), a variable of aerobic exercise capacity, was also assessed. During the examination, abnormalities in blood pressure (BP), heart rate, and electrocardiogram (ECG) were monitored every minute, and the rating of perceived exertion and chest pain scales were measured every 3 min. Relative VO_2_ peak (mL/kg/min), absolute VO_2_ peak (L/min), anaerobic threshold, and exercise duration were recorded using the GXT. The protocol used Bruce, and the termination criterion was RPE 15 or higher and a plateau as long as there were no abnormalities in the clinical indicators or until the participant requested to stop. If the examination could not be completed due to BP, ECG, or chest pain, it was excluded from the analysis. We analyzed anaerobic threshold (AT) as well as VO_2_ peak (mL/kg/min) for relative values and VO_2_ peak (L/min) for absolute values. AT analyzed the ventilation volume of VO_2_ recorded every 10 s in the GXT, found the point where the ventilation volume suddenly increased, and recorded it as a percentage of the relative VO_2_ peak. To calculate metabolic flexibility, VO_2_ and VCO_2_ were recorded for each stage of the Bruce protocol (metabolic equivalents, Mets). The oxidation amount of fat and carbohydrate (CHO) was calculated by applying the formula presented in the Yang et al. study [26].

Grip strength was used as a measure of upper-limb muscle strength [27]. A grip strength meter (TKK 5401, Takei, Niigata, Japan) was used. The participant stood with legs shoulder-width apart, arms extended, and fists placed on the legs. The length of the measurement system was adjusted to fit the middle finger of the patient’s third finger. Following the ‘start’ signal, participants were instructed to clench their fists to the maximum possible extent and not to rest their hands on their legs or move their bodies. This procedure was repeated twice with both hands, and the average of the maximum values was used for the analysis.

For lower-extremity muscle strength, knee extension and flexion were measured using a CSMi isokinetic dynamometer (CSMi HUMAC NORM, Stoughton, MA, USA) [28]. The test was repeated four times at 60°/s, and the maximum value (Newton meter, Nm) was recorded. The examination was explained to the patients, and they were allowed to practice to become familiar with the equipment. A skilled examiner conducted the test, and verbal signals were provided to encourage the exertion of maximal muscle strength during extension and flexion. If the participants were not familiar with the test equipment or a normal graph could not be obtained, a re-test was performed after a break. In the analysis, the extension value was used to measure the muscle strength of the quadriceps [29]. Both sides were tested, and the maximum values of both sides were used for analysis.

### 2.5. SF-36

Quality of life (QoL) was assessed using the SF-36. The SF-36 is a 36-item generic QoL tool with well-established reliability and validity that has been applied extensively in the research and evaluation of health and medical populations [30]. Respondents expressed various health conditions on a Likert scale regarding their physical condition, pain, behavior, and emotions. The SF-36 includes 36 questions comprising physical and mental domains. The patient self-administered the questionnaire using paper and pen, and the assistant researcher provided support only if the patient did not understand the question or requested assistance.

### 2.6. Cardiac Rehabilitation Program and Exercise Prescription

The exercise regimen was based on the guidelines of the ACSM and the American Association of Cardiovascular and Pulmonary Rehabilitation (AACVPR, Chicago, IL, USA) for cardiac patients [25,31]. Karvonen’s formula was used based on the results of the exercise load test, and the exercise intensity was set in the VO_2_ reserve 40–59% range because it was an early outpatient phase [9]. This intensity has a respiratory exchange ratio (RER) of 0.85, calculated as VCO_2_/VO_2_, and is 65–75% when %HRmax is applied. Yang et al. [26] reported that the energy sources oxidized during exercise at this point were approximately 4.73 g/min for carbohydrates and 1.60 g/min for fat. The patients were instructed to perform walking and cycling at speeds that matched the target exercise intensity according to the Rated Perceived Exertion (RPE) scale. Participants were instructed on how to install and use an application for heart rate monitoring. The indicated amount of daily exercise was performed equally by both groups for 60–90 min, including warm-up and cool-down exercises. The CR exercise program consisted primarily of aerobic exercise. High-intensity resistance training with Valsalva maneuvers, which could cause BP elevation, was not included. The researchers designed a program that emphasized safety, being cognizant that the participants were undertaking early CR after coronary artery treatment [32]. The specific training contents are as follows:

Warm-up and cool-down: Static stretching was mainly used, and the stretching to relax large muscle groups was composed of 15–20 motions. In the method, 10–15 s of static stretching was repeated 2–3 times.

Aerobic exercise: Since HFT is a CR center-based training, it was performed for 50–60 min using various equipment. Walking on the treadmill was performed for 30–40 min and biking or using the elliptical trainer for 15–20 min. The LFT was also conducted for 50–60 min like the HFT. When using specialized exercise facilities, the same program as the HFT program was provided. If they use parks and outdoor facilities, they were instructed to walk or cycle.

Light resistance training: In both the LFT and HFT groups, heavy resistance training to the extent that the Valsalva maneuver was induced was prohibited, but large muscle groups were exercised for 10–15 min to manage muscle condition using bodyweight strength training (half squats, crunches), tubing, or light dumbbells (arm curls, lateral raises). The intensity was set below RPE 13 so that the subjective hard intensity did not exceed 50% of the maximum muscle strength.

They were instructed to consume nitroglycerin if chest pain occurred, and counseling was conducted regarding abstinence from alcohol, smoking cessation, and dietary management. Participants were also educated on the recommended daily calorie intake for weight control. A low-fat, low-salt, and low-cholesterol diet was recommended, and menu tables and food models were used to enhance their understanding. All consultations were individual.

### 2.7. Statistical Analysis

Data were analyzed using IBM SPSS Statistics (version 25.0; IBM Corp., Armonk, NY, USA). Shapiro–Wilk tests were conducted to confirm normality. We used a parametric method because blood variables, body composition, and BMI have normality. Meanwhile, since fitness and the SF-36 do not show normality, a non-parametric method was used. Continuous variables were expressed as means and standard deviations using descriptive statistics. Categorical variables were expressed as numbers and percentages and analyzed using the Chi-square test, and the effect size was calculated as cramer’s v. Pre-intervention was expressed as ‘Baseline’ and post-intervention as ‘12-weeks’, and a paired *t*-test was conducted as parameter analysis. Normality variables were subjected to repeated two-way analysis of variance (ANOVA) to determine the effect of interaction by group and time, and effect size was calculated by partial eta squared (partial η^2^; small 0.01, medium 0.006, large 0.14). Non-parametric variables were compared before and after intervention using the Wilcoxon signed rank test. Cohen’s d was used to calculate the effect size for the parametic test and $r=\frac{ɀ}{\sqrt{N}}$ for the non-parametic test. The threshold of the effect size was small 0.2, medium 0.5, and large 0.8 in the parametric test, and small 0.1, medium 0.3, and large 0.5 in the non-parametric test [33]. One-way ANOVA and Bonferroni for post-hoc were performed for fat and CHO oxidation changes over time. Effect size and post-hoc tests were expressed mainly for significant results, with statistical significance set at *p* < 0.05.

## 3. Results

### 3.1. General Characteristics of Participants

Table 1 presents the general characteristics of the participants. Age, height, weight, and BMI were not significantly different between the two groups. There was no significant difference in the patients’ education level and diagnosis; however, there was a significant difference regarding income. The rate of one blood vessel requiring treatment was highest, and the rate of complications associated with three blood vessels was lowest. Most participants were patients with first-time coronary artery disease without recurrence. There was a significant difference between groups in the frequency of training performed by the participants, but no significant difference in training intensity or prescribed medication.

### 3.2. Blood Pressure, Body Composition, Body Mass Index, and Body Composition

Table 2 shows the results for BP and body composition. Systolic BP significantly improved in both groups, and BMI, waist circumference, and body fat percentage exhibited significant changes only in the HFT group. There were interactive effects of time and group regarding systolic BP, BMI, waist circumference, and percent body fat. There was no significant change in skeletal muscle mass in either group.

### 3.3. Lipid Risk Factors for Cardiovascular Disease

**Figure 1:**
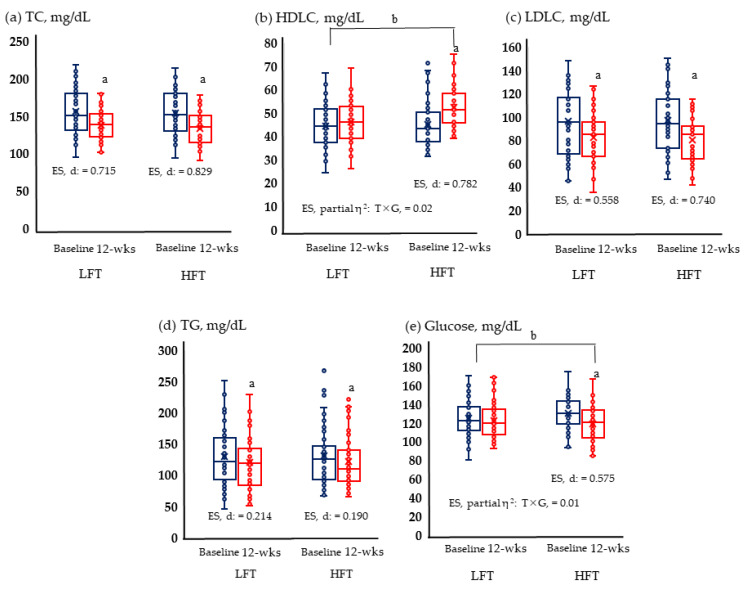
Lipid and glucose metabolism are risk factors for cardiovascular disease; (**a**) Change of TC; (**b**) change of HDLC; (**c**) change of LDLC; (**d**) change of TG; and (**e**) change of glucose in LFT and HFT groups according to 12 weeks intervention. *p* < 0.05; a, Baseline vs. 12 wks; b, time × group; wks, weeks; LFT, low-frequency training; HFT, high-frequency training; ES, effect size; T × G, time × group. TC, total cholesterol; HDLC, high-density lipoprotein cholesterol; LDLC, low-density lipoprotein cholesterol; TG, triglycerides. Blue boxplots are baselines corresponding to pre-intervention, and red boxplots are 12-wks post-interventions.

TC, HDL, LDL, TG, and glucose levels were measured. HFT showed significant improvement in all blood variables. Meanwhile, LFT provided significant changes only in TC, LDLC, and TG, and interaction according to time and group was revealed for HDLC and glucose (Figure 1).

### 3.4. Physical Fitness and Quality of Life

**Figure 2:**
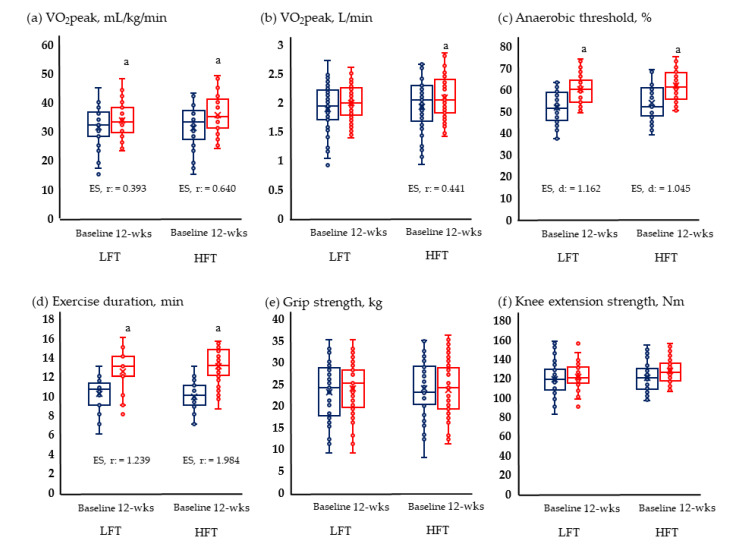
Change in physical fitness; (**a**) Change of relative VO_2_ peak; (**b**) change of absolute VO_2_ peak; (**c**) change of anaerobic threshold; (**d**) change of exercise duration; (**e**) change of grip strength; and (**f**) change of knee extension strength in LFT and HFT groups according to 12 weeks intervention. *p* < 0.05; a, Baseline vs. 12 wks; wks, weeks; LFT, low-frequency training; HFT, high-frequency training; ES, effect size. Blue boxplots are baselines corresponding to pre-intervention, and red boxplots are 12-wks post-interventions.

**Figure 3:**
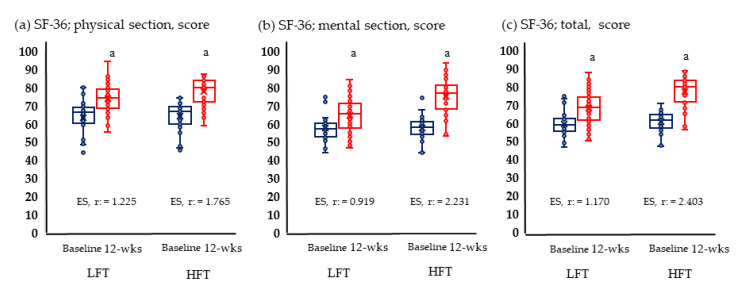
Change in quality of life; (**a**) Change of physical section; (**b**) change of mental section; (**c**) change of total score anaerobic in LFT and HFT groups according to 12 weeks intervention. *p* < 0.05; a, Baseline vs. 12 wks; LFT, low-frequency training; HFT, high-frequency training; ES, effect size. Blue boxplots are baselines corresponding to pre-intervention, and red boxplots are 12-wks post-interventions.

Figure 2 and Figure 3 show the results of physical fitness and quality of life. The GXT results measuring aerobic exercise capacity increased significantly in both groups. Neither group showed significant changes in grip and knee muscle strengths. Quality of life improved significantly in both physical and mental areas in both groups.

### 3.5. Metabolic Flexibility

**Figure 4:**
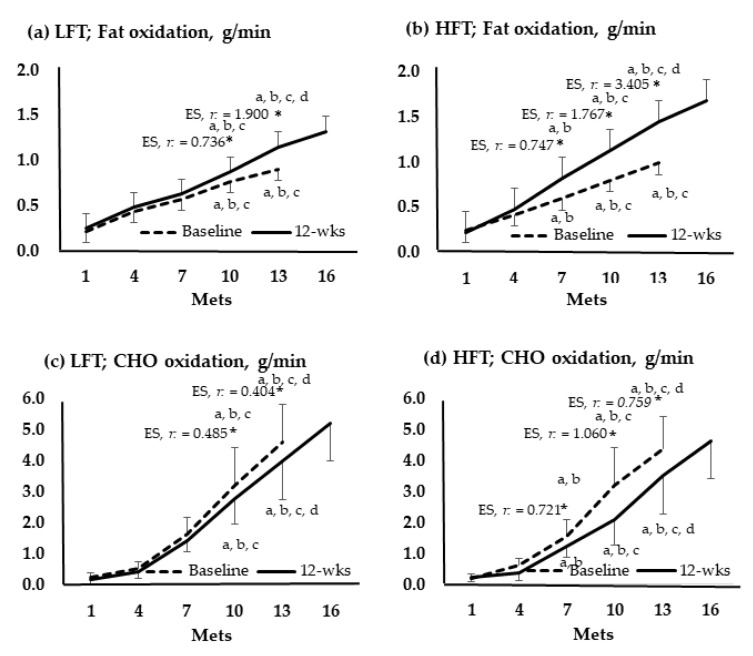
Oxidation of fats and carbohydrates according to the graded exercise test stage. *, *p* < 0.05; a, vs. 1 Mets; b, vs. 4 Mets; c, vs. 7 Mets; d, vs. 13 Mets; Baseline vs. 12-wks; wks, weeks; LFT, low-frequency training; HFT, high-frequency training; Mets, metabolic equivalents; CHO, carbohydrate; ES, effect size.

Figure 4 shows the change in oxidation amounts of fat and CHO by group according to the stage of the GXT. In 10 and 13 Mets of both groups, the amount of fat significantly increased after the intervention compared to before the intervention, and the amount of CHO decreased. In LFT, changes in fat and CHO were not significant at 7 Mets. Meanwhile, at 7 Mets of HFT, fat increased and decreased in CHO.

## 4. Discussion

CR has become a well-established treatment modality for patients with CAD. The effects on physical and psychological recovery, relapse rates, and mortality have been demonstrated [10]. Professional organizations recommend performing aerobic exercise at least three times per week to achieve noticeable clinical effects [25,31]. However, low CR participation rates due to various reasons remain a concern for therapists. Therefore, this study was conducted to confirm the effectiveness of LFT in home-based CR when compared to HFT in center-based CR, although participation was lower than the guideline recommendations.

In terms of body composition, the results of this study exhibited significant changes in body fat, waist circumference, and BMI only in HFT; no significant changes were observed with LFT. A previous study showing results similar to ours reported that the body fat percentage of general adults improved significantly only by performing three sessions per week compared to two sessions per week after eight weeks of training [34]. However, unlike the results of this study, another study reported significant changes even at low frequencies. In a study evaluating 1619 individuals who exercised for 10 weeks, the frequencies of 1, 2, and 3 days per week of activity were compared. Body fat percentage and lean weight significantly changed in both the 2-days-a-week and 3-days-a-week groups [35].

Another study conducted a 60-min exercise program for the elderly for six months and found that exercising two days a week improved both body fat and waist circumference [36]. Conversely, some studies have reported no changes. In a study in which exercise intervention was administered to the elderly for 10 weeks, body fat composition did not improve in either the 2-days-a-week or 3-days-a-week groups [37]. In a similar study, exercising two days a week for 12 weeks showed no improvement in body fat percentage or BMI [38]. Another study reported no change in body composition with exercise once a week [35], and a study showed conflicting results [36]. Body weight and composition are affected by diet; however, the fact that previous studies did not explain diet should be considered in future studies. Additionally, no change in muscle mass occurred during body composition in this study. Although the principle of overload should be applied to create muscle hypertrophy through strength training, we did not subject early-stage CR to heavy resistance training because safety must be guaranteed first [39].

As for BP, only the systolic BP of both groups changed in this study, and these results are different from previous studies. In a study by training frequency, there was no change in BP at 1, 2, and 3 days per week [35], and both systolic and diastolic BP improved in the 1-day and 2-days per week groups [36]. These results were likely influenced by the therapeutic regimen. Since all participants in this experiment were cardiac patients, the effect of drugs affecting BP, such as beta blockers or calcium channel blockers, cannot be ruled out.

One of the key findings of this study was fitness. HFT significantly improved relative and absolute VO_2_ peak values, anaerobic threshold, and exercise time. In LFT, the relative VO_2_ peak, AT, and exercise time were improved. Similar results have been previously reported. The exercise intervention was conducted for heart failure patients for three months: three days a week for the first month, two days a week for the second month, and one day a week for the third month. It has been reported that VO_2_ peak and a six-minute walking ability improved in heart failure patients with greatly reduced exercise capacity [40]. A study involving exercise intervention for older adults for 10 weeks reported that the VO_2_ peak significantly improved in both the 3-days-per-week and the 2-days-per-week groups [37]. Similarly, in a study involving normal adults training for eight weeks, VO_2_ peak, waist circumference, and thigh cross-sectional area increased in the 2- and 3-days-per-week groups compared with the control group, and there was no significant difference between the two groups [34]. In this study, there were no significant changes in grip strength or knee extension strength in any group. Muscle strength was related to muscle mass and showed results similar to those of muscle mass. This may be because the heavy resistance training program was not presented to patients in the early stages of cardiac rehabilitation for safety reasons.

In this study, the TC, LDLC, and TG from the LFT were significantly improved. However, in a previous study, training two days a week for eight weeks had limitations in improving blood variables [34]. These results cannot exclude the possibility of drug and dietary control effects. Many participants were prescribed β-blocker or calcium-channel blocker, hypoglycemic, and antilipemic medications, as well as consulting with clinical nutritionists. In another study, unlike this one, there was an improvement in HDLC even with exercise two days per week [38]. The significant changes in HDLC and glucose observed with HFT in this study are expected to be due to marked improvements in metabolic abnormalities and obesity indicators, such as BMI, waist circumference, and body fat.

In a long-term study conducted for 21 weeks, exercise was performed two days per week by CR patients [41]. There was a partial change at three months, a relatively short period, but mostly no change at 21 weeks. After 12 weeks, only exercise duration and VO_2_ peak changed significantly, and there were no significant changes in weight, lipid variables, or leg muscle strength. Additionally, at 21 weeks, there were significant changes in leg muscle strength and TC but no significant changes in weight, lipid variables, BP, or exercise capacity [41]. These results indicate that while effective changes are observed in the early stages, it is challenging to maintain long-term improvement.

Finally, we also assessed the impact on quality of life. As a result of high-intensity exercise for eight weeks in general adults, both the 2-days-per-week and 3-days-per-week exercise groups exhibited improved physical and mental domains on the SF-36 after the intervention compared to before the intervention [34]. Furthermore, in a study in which water-based training was conducted twice per week for 12 weeks in older adults, improvements were observed in the psychological, environmental, and social quality of life [42]. These results were similar to those of the present study. The improved quality of life provided by exercise has been reported for a long time. CR is effective because it improves physical function through regular exercise participation and resolves anxiety, fear, worry, and irritability caused by cardiac events [43,44].

Although a positive effect of CR has been reported, it is challenging to verify the effect of CR intervention due to the high dropout rate. CR dropout studies have revealed that 12–56% of patients do not complete a CR program [17]. Additionally, an Australian study investigating participation in CR found that the frequency of participating in CR exercise averaged 1.6 days per week and the duration of program participation was only seven weeks. When these individuals participated, the average exercise time per session was 55.5 min, and the exercise intensity was approximately RPE 11–13. According to the principle of frequency–intensity–time (FIT), the exercise was performed to satisfy the guidelines for intensity and time, but the frequency was not satisfied [15]. One study emphasized the importance of frequency. The factors affecting the occurrence of cardiac events were analyzed in a study conducted on patients with type 1 diabetes. The results revealed that the low-intensity group did not display an increased risk compared to the high-intensity group. Similarly, the short-term group did not exhibit a significant increase in risk compared to the long-term group. However, the risk of participation in the low-frequency group increased significantly by a factor of 1.69 compared to that in the high-frequency group [45].

This study includes the contents to be discussed in relation to exercise prescription. The method used to set the target exercise intensity in our study is the Karvonen formula. Unlike %HRmax, this method is characterized by including the resting heart rate in the formula. However, the exercise target heart rate using this formula has the disadvantage of high variation. This is because the resting heart rate may not be stable at the beginning of treatment or due to arrhythmia, and the β-blocker or calcium-channel blocker drugs that most heart patients take greatly affect the change in heart rate. Thus, the Karvonen formula may overestimate or underestimate the exercise intensity of a particular patient [46]. Therefore, if the target exercise intensity applied in this study had been underestimated, it might not have been of sufficient intensity to change BMI, waist circumference, body fat, HDLC, and glucose in the LFT group.

This study was conducted at low intensity. The advantage of low-intensity exercise is that long-term exercise is possible because the rate of fat oxidation is higher than that of carbohydrates and the accumulation of lactate is less. During low-intensity aerobic exercise, energy is supplied by fat oxidation rather than carbohydrates. According to Yang et al. [47], they reported that it is efficient and that a small amount of lactate in the blood generated from glycolysis due to fat oxidative metabolism is re-synthesized and used as an energy source. Therefore, it can be said that low-intensity exercise is better for recovery and reproduction.

In this study, changes in metabolic flexibility were confirmed, which means that fat and CHO, which are energy sources, change efficiently according to exercise intensity, and this study analyzed them using the oxidation amount calculation formula.

Both groups showed a decrease in CHO and an increase in fat oxidation. This translates into increased efficiency through increased use of fat rather than CHO at the 10 and 13 Mets. This phenomenon is similar to the results of athletes using more fat and having lower CHO oxidation than non-athletes at the same intensity. In addition, the alternation between fat oxidation and lactate concentrations appeared at a faster rate in athletes than in general populations [26]. Moreover, there was no change in metabolic flexibility at 7 Mets of LFT, which is probably because 1–2 training sessions were not able to provide sufficient stimulation. The low-intensity aerobic exercise performed by the participants in this CR would have enabled continuous energy supply by inducing high fat oxidation by promoting gluconeogenesis in triglycerides and, therefore, would have induced significant changes in glucose. 

Ultimately, in this study, participation in low-intensity CR resulted in increased fat metabolism efficiency. CHO metabolism is not necessarily absolute in high-intensity training. In the Yang et al. study [48], even in the high-intensity state of fencers, the energy contribution was supplied by oxidative contribution rather than the ratio of phosphagen and glycolysis, and this phenomenon was found to be higher in the second and third bouts than in the first bout.

Metabolic flexibility changes are not observed only in skeletal muscle. Changes in metabolic flexibility also appear in the heart muscle, one of the representative muscles in the body. The energy substrates that contributed to the heart were 83% free fatty acid, 4% glucose, and 13% lactate at rest, but at VO_2_ max 40%, it decreased to 40% free fatty acid and 4% glucose, and lactate increased to 56% [49]. Although this study was conducted on middle-aged women, Lewsey et al. [50] reported that frail elderly people experience rapid high-energy phosphate metabolism decline. In future studies, the topic of energy contribution as a CR effect for various age groups should be added.

In this study, we trained patients to self-monitor their heart rate using a smartphone application. Due to the recent popularization of smart phones, various CR studies using mobile healthcare systems have been conducted [19,51,52]. Overall, similar positive results have been reported when compared to supervised training, and experts predict that the role of mobile health care services will expand.

Compared to the increasingly high CAD in women, low CR and physical activity in women are becoming an increasingly important problem [53,54]. Experts offer various opinions about women’s low CR referral, enrollment, and completion rates. In particular, patients’ lack of CR awareness is a major cause of the low participation rate [55]. In addition, low insurance status, low economic status due to unemployment, and high rates of complex diseases suggest that women have more difficulty accessing CR than men. In order to solve these difficulties, not only doctors but also the support of family and friends is emphasized [10].

The main information provided by the results of this study is similar to that found in the existing literature. The high-frequency CR participants showed more positive effects than the low-frequency participants. However, some patients may have difficulty participating in exercise for more than three days per week for various reasons. The results of this study reported on the benefits of home-based LFT. This information will be useful for early CR patients who cannot visit the center.

Despite its usefulness, this study had the following limitations: Dietary counseling was conducted, but analyses based on nutritional assessments were not included. Therefore, the results may differ in terms of body composition, obesity, and blood variables that are influenced by diet. In addition, since this study was conducted on middle-aged women, the results may not be generalizable for men and older adults. Participants in menopausal transition and menopause were included because they were middle-aged women, but detailed classification was not performed in this study. In particular, this study did not consider the fact that hormonal changes after menopause have physiological, psychological, and physical effects [56]. The study was conducted at a single institution, and the various morbid conditions of patients were not considered. Group assignments were conducted according to the patient’s preference and circumstances (e.g., time of visits and distance to the center). Finally, one of the major limitations of this study is that there is a significant difference in household income for HFT compared to LFT. Studies of socioeconomic status and health status have shown that higher incomes have higher access to medical care and better health care status, thus increasing the likelihood of having a healthy diet and high levels of physical activity [57,58]. In the future, a randomized case-controlled study should be performed. A future study that augments our current evidence and addresses these limitations will add significant value to CR.

## 5. Conclusions

A 12-week CR exercise program was conducted for middle-aged women who underwent stent implantation and participated in early-stage CR. The HFT center-based group and the LFT home-based group were compared. All blood variables in HFT were improved, but only TC, LDLC, and TG were improved in LFT. HFT was more effective than LFT in improving BMI, waist circumference, body fat, weight, HDLC, and glucose. Meanwhile, both LFT and HFT had significant effects on the improvement of relative VO_2_ peak, anaerobic threshold, exercise duration, and quality of life. In addition, in both groups, the energy contribution through oxidation was increased in fat and decreased in CHO at the same intensity, and metabolic flexibility was improved. Therefore, participation in CR at the guideline level is most recommended for CAD patients, but participation once or twice a week should be a sub-optimal option for patients who have difficulty participating sufficiently for various reasons.

## Figures and Tables

**Table 1:** Characteristics of participants according to CR attendance frequency.

Variables	LFT (*n* = 45)	HFT (*n* = 45)	*t* or χ-Value	Cohen’s d or Cramer’s v	*p*-Value
Age, years	51.7 ± 6.3	50.6 ± 5.7	0.115	0.199	0.788
Height, cm	162.7 ± 3.4	163.2 ± 4.6	1.096	0.123	0.275
Weight, kg	60.3 ± 7.7	61.6 ± 6.9	0.356	0.177	0.723
BMI, kg/ m^2^	22.8 ± 2.9	23.1 ± 2.3	−0.363	0.114	0.718
Vessels, *n* (%)					
One	28 (62.2)	31 (68.9)	0.730	0.084	0.730
Two	15 (33.3)	13 (28.9)			
Three	2 (4.5)	1 (2.2)			
Recurrence, *n* (%)					
Yes	3 (6.7)	2 (4.4)	0.645	0.049	0.639
No	42 (93.3)	43 (95.6)			
Education, *n* (%)					
Middle	5 (11.1)	3 (6.7)	0.720	0.089	0.698
High	18 (40.0)	17 (37.8)			
College	22 (48.9)	25 (55.5)			
Income, per year, USD, *n* (%)					
<5000	24 (53.3)	13 (28.9)	11.299	0.354	0.004
5000–10,000	18 (40.0)	17 (37.8)			
>10,000	3 (6.7)	15 (33.3)			
Diagnosis					
Unstable angina	39 (86.7)	35 (77.8)	1.216	0.116	0.409
Myocardial infarction	6 (13.3)	10 (22.2)			
Training frequency, days/week	4.6 ± 0.3	4.3 ± 0.6	0.258	0.632	<0.001
Training intensity, RPE scale	12.5 ± 1.1	13.1 ± 0.8	1.105	0.623	0.105
Medication information					
β-blocker or calcium-channel blocker	38 (84.4)	35 (77.8)	1.111	0.111	0.430
Antilipemic medication	33 (73.3)	36 (80.0)	0.241	0.052	0.807
Hypoglycemic medication	7 (15.6)	10 (22.2)	0.653	0.085	0.591

*p* < 0.05; LFT, low-frequency training; HFT, high-frequency training; BMI, body mass index; USD, United States dollar; RPE, rated perceived exertion.

**Table 2:** Comparison of blood pressure, body mass index, and body composition.

Variables	Group	Baseline	12-wks	Df, %	*p*-Value	Cohen’s d	T × G Partial η^2^	T × G *p*-Value
SBP, mmHg	LFT	128.0 ± 17.2	122.2 ± 16.6	−4.5	0.041	0.343	0.029	0.014
	HFT	129.2 ± 15.3	118.8 ± 14.8	−8.0	0.017	0.690		
DBP, mmHg	LFT	76.0 ± 11.1	74.8 ± 10.3	−1.6	0.612	0.112	-	0.518
	HFT	75.2 ± 9.4	74.5 ± 12.2	−0.9	0.404	0.040		
Weight, kg	LFT	60.3 ± 7.7	59.9 ± 6.4	−0.7	0.550	0.056	0.031	0.005
	HFT	61.6 ± 6.9	58.3 ± 5.8	−5.4	0.011	0.517		
BMI, kg/m^2^	LFT	22.8 ± 2.9	22.6 ± 2.1	−0.5	0.178	0.078	0.025	0.009
	HFT	23.1 ± 2.3	21.9 ± 2.0	−5.2	0.015	0.556		
Waist circumference, cm	LFT	94.7 ± 6.3	92.4 ± 5.8	−2.4	0.302	0.091	0.037	0.035
	HFT	95.3 ± 6.8	91.5 ± 6.6	−4.0	0.041	0.588		
Body fat, %	LFT	27.1 ± 5.4	24.2 ± 4.2	−10.7	0.133	0.599	0.015	0.020
	HFT	27.9 ± 5.1	23.7 ± 4.6	−15.1	0.011	0.864		
Skeletal muscle mass, %	LFT	26.1 ± 2.4	26.7 ± 2.6	2.3	0.314	0.239	-	0.608
	HFT	26.5 ± 2.3	27.0 ± 3.1	1.9	0.263	0.183		

*p* < 0.05; wks, weeks; Df, difference; LFT, low-frequency training; HFT, high-frequency training; SBP, systolic blood pressure; DBP, diastolic blood pressure; BMI, body mass index; T × G, time × group.

## Data Availability

The data are not publicly available because of privacy or ethics.
